# Identification of Novel QTL Conferring Sheath Blight Resistance in Two Weedy Rice Mapping Populations

**DOI:** 10.1186/s12284-020-00381-9

**Published:** 2020-03-23

**Authors:** David M. Goad, Yulin Jia, Andrew Gibbons, Yan Liu, David Gealy, Ana L. Caicedo, Kenneth M. Olsen

**Affiliations:** 1 Department of Biology, Washington University in St. Louis, 1 Brookings Drive, Campus Box 1137, St. Louis, MO 63110 USA; 2 United States Department of Agriculture-Agricultural Research Service, Dale Bumpers National Rice Research Center, 2890 HWY 130 E, Stuttgart, AR 72160 USA; 3 University of Arkansas Rice Research and Extension Center, 2900 AR-130, Stuttgart, AR 72160 USA; 4 Present address: Arkansas Department of Health, Little Rock, AR 72205 USA; 5 Present address: Department of Plant Pathology, Washington State University, Pullman, WA 99164 USA; 6 Department of Biology, University of Massachusetts, Amherst, USA

**Keywords:** Quantitative trait loci, Sheath blight disease, Rice, Genotyping-by-sequencing, Weeds

## Abstract

**Background:**

Rice sheath blight (ShB) disease, caused by the pathogenic fungus *Rhizoctonia solani*, causes significant yield losses globally. US weedy rice populations, which are de-domesticated forms of *indica* and *aus* cultivated rice, appear to be more resistant to ShB than local *japonica* cultivated rice. We mapped quantitative trait loci (QTL) associated with ShB resistance using two F_8_ recombinant inbred line populations generated from crosses of an *indica* crop variety, Dee-Geo-Woo-Gen (DGWG), with individuals representing the two major US weed biotypes, straw hull (SH) and black hull awned (BHA).

**Results:**

We identified nine ShB resistance QTL across both mapping populations. Five were attributable to alleles that affect plant height (PH) and heading date (HD), two growth traits that are known to be highly correlated with ShB resistance. By utilizing an approach that treated growth traits as covariates in the mapping model, we were able to infer that the remaining four QTL are involved in ShB resistance. Two of these, *qShB1–2* and *qShB4*, are different from previously identified ShB QTL and represent new candidates for further study.

**Conclusion:**

Our findings suggest that ShB resistance can be improved through favorable plant growth traits and the combined effects of small to moderate-effect resistance QTL. Additionally, we show that including PH and HD as covariates in QTL mapping models is a powerful way to identify new ShB resistance QTL.

## Background

Rice sheath blight (ShB) disease, caused by the soil borne fungus *Rhizoctonia solani* (teleomorph: *Thanatephorus cucumeris*), is one of the most devastating pathogens of rice worldwide (Savary et al. 2006). In the United States, it can cause up to 50% yield loss in infected fields (Rush and Lee 1983). The pathogen infects the plants at the waterline and spreads upwards, creating lesions on the leaf blades and sheaths. Infection to any detectable degree reduces yield, but the fungus is particularly destructive if it reaches the panicle and infects the grains. Because of its worldwide impact, there have been many attempts to identify genes in rice that confer increased ShB resistance. However, to date, few major ShB resistance genes have been identified from either cultivated rice or wild rice relatives (Molla et al. 2019). Over 25 quantitative trait locus (QTL) mapping studies have been performed using crosses from a diverse set of parents, including improved cultivars (Liu et al. 2009; Li et al. 1995; Zou et al. 2000), landraces (Xu et al. 2011; Taguchi-Shiobara et al. 2013), and the wild rice species *Oryza nivara* (Eizenga et al. 2013) and *O. meridonalis* (Eizenga et al. 2015)*.* These studies have produced a wealth of putative small-effect QTL; however, only a few of these loci, such as *qSB-9*^*TQ*^ (Zuo et al. 2014) and *qSB-11*^*LE*^ (Zuo et al. 2013) have been fine mapped and used for breeding. There is an urgent need to manage ShB by identifying novel sources of disease resistance and implementing them in management or breeding programs.

Mapping traits in new and diverse populations is one of the best ways to identify additional resistance QTL. So far, weedy rice accessions have not been used in developing ShB-resistant mapping populations despite their potential to harbor unique resistance alleles (Liu et al. 2015). Weedy rice is often the result of de-domestication of cultivated forms of rice (*Oryza sativa*) that occurs in rice production areas worldwide and aggressively outcompetes its domesticated relative (Wedger and Olsen 2018). In the US, two major biotypes of weedy rice are most common; they both have a red pericarp, but are largely distinguishable by grain hull characteristics and are referred to as straw hull (SH) and black hull awned (BHA) types (Londo and Schaal 2007). The SH and BHA weed biotypes are genetically distinct (Londo and Schaal 2007) and have been shown to have evolved independently by de-domestication from cultivated Asian rice varieties (SH from *indica* rice, BHA from *aus* rice) (Reagon et al. 2010; Li et al. 2017). Several factors suggest that these weeds could be promising sources of ShB resistance genes such as: 1) The SH and BHA strains are among the most predominant weeds in southern US rice fields where sheath-blight is the most destructive pathogen (Wrather and Sweets 2009), which suggests that they may possess a mechanism of disease resistance that confers a competitive advantage. 2) Weed **×** crop mapping populations for both biotypes have already been used to identify resistance QTL for another fungal disease (rice blast) (Liu et al. 2015). 3) Finally, because the two weed biotypes evolved independently and have historically shown limited hybridization with US cultivated rice (Reagon et al. 2010), any resistance alleles that they carry are likely to be unique to the weeds and unlikely to have been previously identified.

A complication in ShB-resistance genetic mapping studies is that the level of infection shown by a plant is correlated not only with resistance directly interacting with the pathogen but also with plant growth traits, particularly plant height (PH) and heading date (HD) (Li et al. 1995; Zou et al. 2000; Channamallikarjuna et al. 2010). ShB resistance has been typically scored on a 0–9 or 1–9 scale by measuring the proportion of the stem above the waterline with signs of infection with 1 being very resistant and 9 being very susceptible. With this scoring system, greater PH is directly correlated with a higher resistance score (Li et al. 1995). In the case of HD, the cause of the correlation is less well understood. It has been suggested that later-heading varieties are more resistant because they grow later in the season, when conditions are drier and less favorable for pathogen spread (Wasano and Hirota 1986), but this hypothesis has not been formally tested. While these growth traits may be of interest for selecting varieties in areas with severe ShB, they confound attempts to genetically map QTL for ShB resistance. Indeed, in most previous mapping studies of ShB resistance, the largest effect QTL have been directly attributable to loci for either PH or HD (as reviewed by Zeng et al. 2015). The confounding effects of growth traits with resistance could be particularly problematic for weed **×** crop mapping populations, as weedy rice differs dramatically from cultivated rice in both traits. However, it is possible to factor out these confounding effects if QTL mapping models that explicitly incorporate PH and HD measurements into the analysis as covariates are employed. Despite the potential utility to detect QTL associated with resistance, covariate modeling has been underutilized in the ShB QTL mapping literature, with (Nelson et al. 2012) as the sole example of this strategy.

In this study, we map QTL associated with ShB resistance using two weed **×** crop F_8_ mapping populations (derived from SH **×** crop and BHA **×** crop crosses) that were assessed in field conditions over two growing seasons. By using PH and HD measurements as covariates, we were able to remove the effects of QTL associated with these confounding growth traits and identify novel ShB resistance QTL and interactions that would otherwise have been undetected. Our SNP linkage maps for these populations yielded greater genetic resolution than previous mapping studies and have allowed us to identify potential candidate genes within our QTL confidence intervals. The relationship between PH, HD, and ShB confirmed in this study also has implications for optimal cultivar choice in regions of high ShB incidence and management practices in fields with recurring infestations.

## Methods

### Inoculum Preparation

To produce the required amounts of *R. solani*, a slow growing field isolate (RR0134) was chosen. The isolate was grown on a potato dextrose agar (PDA) by introducing shredded mycelium-infiltrated filter paper to the culture plate. The plate was incubated at 30 °C until the appearance of black-bodied sclerotia. This product was used as the initial inoculants. To grow large amounts of *R. solani*, a mixture of corn, rye, and water in the proportion of 2.48 kg:1.27 kg: 3.5 to 3.75 l (L), respectively was mixed and allowed to soak for 30 min. The mixture was then autoclaved for 1 h at 121 °C/1.0 kg/cm^2^. After the media were allowed to cool overnight, they were mixed and double-bagged. The double-bagged media were loosely sealed and autoclaved an additional two cycles (1 h/121 °C/1.0 kg/cm^2^). The sterilized media were then transferred into 42 cm × 20 cm × 16 cm (11.4 L) plastic containers and allowed to cool prior to inoculation. Each container with the corn/rye media were inoculated by cutting the PDA media containing *R. solani* into 1 to 2 cm squares. The PDA squares were transferred into the sterilized mixture and the tubs were covered with a lid and placed in a growth environment of 25-30 °C and 45% relative humidity. The fungi were grown in the sterilized mixture for 3–5 days until the presence of white-bodied sclerotia were noted. The media containing sheath blight pathogen were then air dried and ground. A total of 90 kg of inoculation media was produced and used for inoculation for each year.

### Plants and Data Collection

Advanced-generation recombinant inbred line (RIL) lines derived from two weed **×** crop crosses were used in the study. The two weedy rice parents of the mapping populations were an *indica*-like straw hull accession (PI 653435; also known as AR-2001–1135-01, and RR9) and an *aus*-like black hull awned accession (PI 653419; also known as MS-1996-9, and RR20). The two varieties were crossed with the *indica* landrace accession Dee Geo Woo Gen (DGWG) to produce two F_2_ mapping populations by (Thurber et al. 2013). These lines were then self-fertilized for 6 generation to create two F_8_ RIL populations totaling 184 lines from the SH **×** DGWG cross (referred to hereafter as the S population) and 236 lines from the BHA **×** DGWG cross (hereafter, the B population). Genotyping of the RILs occurred in the F_5_ generation as described previously (Qi et al. 2015). In the 2015 and 2016 field season each RIL was planted with drill sowing in three rows that were 1.5 m long with a 0.6 m alley separating them. Each line was planted in three replicates in a complete block design where each block was one replication. The cultivar Lemont was planted as a border.

Following the protocol of Liu et al. 2013, approximately 50 g of *R. solani* (AG1-IA, teleomorph: *Thanatephorus cucumeris*) was spread per line along the bottom of the middle row in between the plant tillers at the water plant interface, 72 days after planting. Twenty-four days after inoculum was spread when the plants were in the heading and early flowering stages, each line was rated on a 1–9 scale. The entire middle row of plants was observed from the base of the plant to the panicle. For every 10% of the plant covered in lesions, the score was increased by an increment of 1 (e.g. 10% =1, 20% = 2, …, 90% =9). A line was also scored as a 9 if the infection reached the panicle. PH and HD phenotypes were obtained from the 2012 field season. PH was measured at 100 days after emergence. HD measurements were obtained from (Qi et al. 2015).

### Genotyping

Modified versions of the F_5_ linkage maps for both populations generated by Qi et al. 2015 were used in this study. To remove problem markers we filtered SNPs if they had > 10% missing data or minor allele frequency < 20%. Additionally SNP positions were updated from the MSU 6.0 genome assembly to their MSU 7 positions using an in-house script. Final SNP counts are 4733 for the S population and 11,853 for the B population.

### QTL Analysis

Analyses were performed in R with the *R/qtl* package (Broman et al. 2003) using a forward stepwise model fitting method with the Haley-Knott algorithm (Haley and Knott 1992). QTL were considered significant if their LOD score was higher than 3. Analyses for each year and mapping population were first performed without phenotypic covariates. Then each analysis was repeated using PH and HD as covariates. All possible interactions between QTL and covariates were tested. Any interactions above LOD 2 were included in the model because the risks associated with multiple hypothesis testing are lower with fewer comparisons. Moreover, identifying a false positive interaction between QTL that are known to be significant is less problematic than including a QTL that is actually a false positive. QTL positions were refined using the *refineqtl* function. The final LOD score and effect size of each QTL were calculated using a drop-one analysis within the *fitqtl* function. The 1-LOD support interval was calculated and visualized using the *r/qtltools* functions *calcCis* and *segmentsOnMap* respectively (Lovell 2017). Regions within the 1-Lod support interval of a QTL were searched for functionally characterized R genes in the Q-TARO database (Table S1) (Yamamoto et al. 2012).

### Phenotypic Analyses

For both populations, the correlations between the 2015 and 2016 measures of ShB susceptibility were calculated using the lm function in R. A model including the phenotypic covariates (and their potential interactions) in the absence of any genotypic markers was created for each population and year. The PVE and LOD for the full model and each individual phenotype and interaction were determined using the drop-one analysis within the *fitqtl* function.

## Results

### Phenotypes

**Fig. 1:**
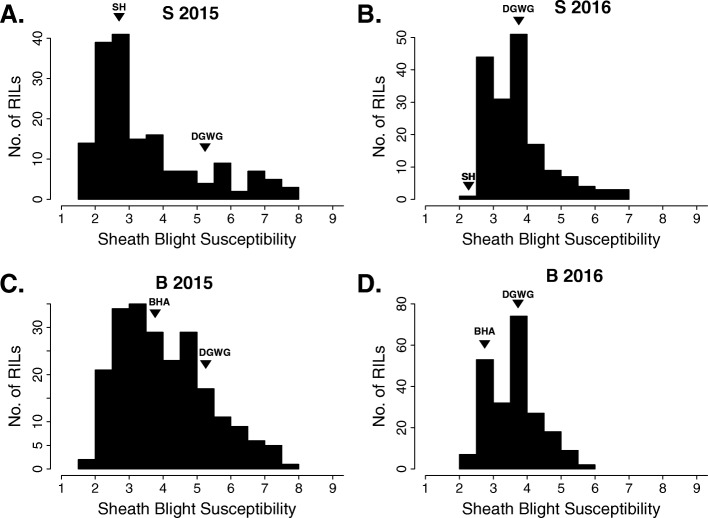
Distribution of sheath blight susceptibility phenotypes in two F_8_ RIL populations. Histograms are shown for the S population in 2015 (**a**) and 2016 (**b**), and the B population in 2015 (**c**) and 2016 (**d**). Susceptibility was measured on a 1–9 scale from least to most infected. The phenotypes of the parents of each population are marked with arrows. DGWG = Dee-Geo-Woo-Gen, BHA = Black-hull awned, SH = Straw-hull

Raw phenotypic data for the B and S populations are given in Table S2 and Table S3, respectively. The distribution of ShB resistance in each year and mapping population is presented in Fig. 1a-d. Sheath blight resistance was correlated between the 2015 and 2016 field season in both the B and S population (*R*^2^ = 0.25, *p* < 0.001; and *R*^2^ = 0.47, *p* < 0.001 respectively). Both weed parents showed lower susceptibility than the crop parent. The cultivated parent DGWG had an average ShB susceptibility score of 5.3 in 2015 and 3.7 in 2016. The BHA parent had scores of 3.5 and 2.9 in 2015 and 2016, respectively. The SH parent scored 2.5 in 2015 and 2.3 in 2016. For the RILs, the average ShB susceptibility scores for the B population were 4.1 and 3.7 in 2015 and 2016, respectively. The average scores for the S population were 3.6 and 3.8 in 2015 and 2016, respectively. Transgressive segregation was more prevalent in 2015 for both populations. The B population had 80 RILs more resistant than BHA (36% of all lines) and 36 more susceptible than DGWG (16% of all lines). The S population had 36 RILs more resistant than SH (21% of all lines) and 27 more susceptible than DGWG (16% of all lines). The genotypes of the 10% most resistant RILs in 2015 for each population at each of our putative QTL are presented in Table S4; these genotypes may be of particular interest for breeding.

The combined effects of PH and HD explain 37.6% of the ShB variance in the B population in 2015 and 32.8% in 2016 (Table 1). There was no significant interaction between the two growth-related phenotypes in either year (*p =* 0.16; and *p* = 0.76 in 2015 and 2016 respectively). In the S population, a model consisting of PH, HD and their significant interaction explained 58.2% of the ShB resistance variance in 2015 and 51.3% in 2016 (Table 1).

**Table 1:** Results of models explaining variance in sheath blight resistance using only phenotypic covariates. The full model represents the additive effects of plant height, heading date and any significant interaction. The statistics for each individual component of the model are based on a drop-one analysis

Dataset	Model components	LOD^a^	PVE(%)^b^
B 2015	Full model	22.4	37.6
	Plant height	14.3	22.0
	Heading date	8.6	12.3
B 2016	Full model	18.9	32.8
	Plant height	16.5	27.8
	Heading date	2.1	3.0
S 2015	Full model	32.1	58.2
	Plant height	31.4	56.6
	Heading date	7.5	9.5
	Plant height x Heading date	5.4	6.6
S 2016	Full model	26.6	51.3
	Plant height	22.5	40.8
	Heading date	14.7	23.7
	Plant height x Heading date	6.8	9.8

^a^Logarithm of Odds

^b^Percent of variance explained

### QTL Analysis

#### B Population

**Fig. 2:**
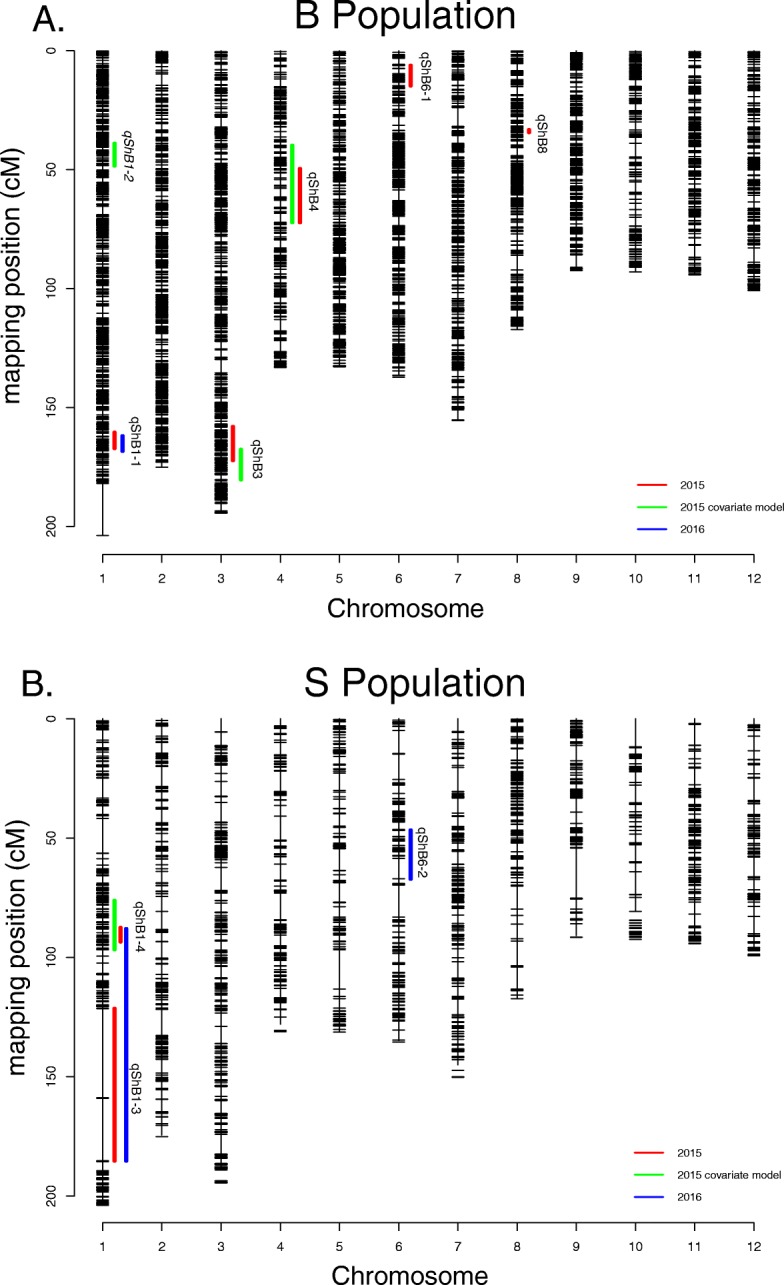
High-resolution genetic map of the B population (**a**) and S population (**b**). Vertical lines represent each chromosome and horizontal dashes represent SNP markers. Genetic distances are measured in centimorgans. Bars represent the 1-LOD confidence interval of a QTL. Color indicates the year and mapping model. There were no significant QTL in 2016 for the model with covariates

Raw genotype data for the B population can be found in Table S5. Figure 2a shows the 1-LOD support intervals for both models in each year for the B population. When the QTL analysis was performed without phenotypic covariates using the 2015 ShB resistance data, five QTL were identified (indicated by red bars in Fig. 2a). Two of these, *qShB1–1* and *qShB8*, had very large effects on the phenotype, with the weedy allele conferring an increased resistance at both loci (Table 2). However, both of these QTL co-localized with known PH and HD genes *sd1* and *DTH8*, respectively (Sasaki et al. 2002; Wei et al. 2010), and both disappeared when the analysis was performed using PH and HD as covariates. A small-effect QTL (*qShB6–1*) also co-localizes with a HD QTL previously identified in the B population (Qi et al. 2015) and was also removed. With the PH and HD QTL factored out, two small-effect ShB QTL remained significant (*qShB3* and *qShB4*) and one new QTL was detected on chromosome 1 (*qShB1–2*) (indicated in green; Fig. 2a). Notably, each of these three putative QTL contained at least one functionally characterized R-gene. The loci *qShB4* and *qShB1–2* contained *OsSERK1* and *OsRac1*, respectively, while *qShB3* contained both *OsCPK10* and *phyC*. Each of these genes is annotated as being involved in blast disease resistance (Ono et al. 2001; Hu et al. 2005; Xie et al. 2011; Fu et al. 2013). A weak interaction between *qShB1–2* and *qShB4* was found, as well as a moderate interaction between *qShB3* and HD. The only QTL identified in the 2016 data was *qShB1–1* (indicated in blue; Fig. 2a); it was significant when plant growth covariates were included, as it did for the 2015 data.

**Table 2:** Sheath blight resistance QTL identified in the two mapping populations in 2015 and 2016

Model	QTL or covariate^a^	Chromosome	LOD^b^	PVE(%)^c^	Effect	Allele with increased resistance	1-LOD start^d^	1-LOD stop^d^	Candidate gene
B population									
2015	qShB1–1	1	14.26	18.26	−0.61	weed	37,154,608	38,207,165	*sd1*
	qShB8	8	9.61	11.71	−0.46	weed	4,097,722	4,604,002	*DTH8*
	qShB3	3	4.60	5.32	0.32	crop	30,382,168	33,046,007	
	qShB4	4	4.50	5.19	−0.31	weed	20,697,856	23,609,792	
	qShB6–1	6	3.59	4.10	0.28	crop	2,046,269	3,171,640	*Hd3a*
2015 with covariates	PH		17.94	21.55	−0.03				
	HD		11.70	13.11	−0.06				
	qShB3	3	7.02	7.48	0.38	crop	33,657,106	35,750,342	
	qShB1–2	1	5.71	6.00	−0.21	weed	7,173,743	38,433,096	
	qShB4	4	3.71	3.82	−0.18	weed	20,697,856	23,609,792	
	qShB1–2 x qShB4		2.30	2.34	0.21				
	qShB3–1 x HD		3.65	3.68	−0.03				
2016	qShB1–3	1	13.27	24.06	−0.39	weed	37,310,074	38,481,437	
S population									
2015	qShB1–3	1	12.83	24.40	−0.84	weed	28,586,738	40,526,762	*sd1*
	qShB1–4	1	3.12	5.16	−0.38	weed	22,783,068	23,642,407	
2015 with covariates	PH		32.29	53.03	−0.24				
	HD		7.69	8.76	−0.24				
	qShB1–4	1	3.86	4.17	−0.33	weed	20,454,249	24,016,179	
	PH x HD		5.65	6.26	0.00				
2016	qShB1–3	1	5.99	13.76	−0.34	weed	22,920,401	40,526,762	*sd1*
	qShB6–2	6	5.02	11.39	0.32	crop	8,057,485	16,481,013	*hd1*

^a^PH, Plant height; HD, Heading date

^b^LOD, Logarithm of Odds

^c^PVE, Percent of the phenotypic variance explained

^d^Genomic position based on MSU7 assembly

#### S Population

Raw genotype data for the S population can be found in Table S6. Two QTL were identified in the S population on chromosome 1 in the model without covariates for the 2015 ShB resistance data (*qShB1–3* and *qShB1–4*) (indicated in red; Fig. 2b). When PH, HD, and their interaction were included as covariates, *qShB1–3* was no longer significant while *qShB1–4* remained significant (indicated in green; Fig. 2b). For the 2016 field data, one new QTL was discovered (*qShB6–2*) (indicated in blue; Fig. 2b). This is a large-effect QTL that was only identified in the model without covariates and which contains *hd1,* a known HD gene (Yano et al. 2000). The single putative resistance QTL (*qShB1–4*) contained one functionally characterized fungal R-gene, *OsACO7*, which is annotated as a blast resistance gene (Iwai et al. 2006).

## Discussion

Identifying QTL associated with increased resistance to ShB is of vital importance for increasing rice yields worldwide. One of the most challenging aspects of this search is to identify loci conferring resistance to the disease as contrast to those reflecting correlated growth traits (PH and HD). Our explicit incorporation of these confounding traits as covariates in the mapping model allowed us to accurately infer how each QTL contributes to ShB resistance and identify putative resistance QTL for further study. Additionally, the SNP linkage maps used in our analyses allowed us to identify functionally characterized candidate genes within each of the QTL. The close relationship between PH, HD, and ShB resistance that we confirm in this study also has implications for ShB management.

### ShB QTL Explained by Plant Height or Heading Date

The observed difference in ShB resistance between DGWG and weedy rice appears to primarily be explained by loci controlling PH and HD. This is not surprising, given previous work demonstrating differences in PH and HD between the parents of the mapping population (Reagon et al. 2011; Thurber et al. 2013; Qi et al. 2015). In both the B and S mapping populations, the single greatest contributor to sheath blight resistance was a QTL located on chromosome 1 (*qShB1–1* and *qShB1–3* respectively). This QTL contains the famous green revolution gene *semidwarf1* (*sd1*), and the influence of this locus was removed in both populations when PH was used as a covariate. Our crop parent, DGWG, is the source of the original *sd1* allele that confers reduced plant height without loss of yields, whereas both weed parents have the wild-type, non-dwarf allele (Reagon et al. 2011; Thurber et al. 2013; Li et al. 2017). Therefore, it is reasonable to assume that this QTL is only indirectly related to ShB resistance due to its strong effect on PH.

Both the B and S population possessed unique QTL for ShB resistance that appear to be due to effects on HD. All three of these QTL contained HD genes known to be involved in the HD difference between the crop and weed parents. Specifically, *qShB6–2* contains *hd1* which has a loss of function mutation in the weed, *qShB8* contains *DTH8* which has a loss of function mutation in the crop parent, and *qShB6–1* contains *Hd3a* which has variation in the promoter that is associated with HD in both weeds and crops (Thurber et al. 2013, 2014; Qi et al. 2015). The PH QTL can also be assumed to be only indirectly related to ShB resistance, in this case, through their effect on HD.

### QTL Conferring Resistance to ShB

Of the loci that remained significant after including PH and HD as covariates, *qShB3* had the highest LOD score (8.1) and explained the largest percentage of the phenotypic variation (8.6%) (Table 2). This QTL was only significant in the B mapping population, and the weed allele conferred reduced resistance. It is located near a previously identified ShB resistance QTL in other mapping populations, suggesting that the DGWG allele, which confers increased resistance, may be present in other germplasm (Zou et al. 2000; Liu et al. 2009). The locus *qShB1–4* from the S population may also overlap with a previously identified QTL (Channamallikarjuna et al. 2010; Jia et al. 2012). Notably, it does not overlap with *qShB1–2* from the B population. It is difficult to make exact position comparisons between linkage maps generated using SNP markers and earlier maps based on SSRs, so it is impossible to say for certain that these QTL represent those found in the prior studies. Even if they do overlap, however, it is still possible that the alleles responsible for increased resistance are unique to our study because of the divergence between weedy rice and the parents of the previous mapping population and germplasm used for genome wide association studies. The remaining two QTL (*qShB1–2* and *qShB4*) have small effects but both provide resistance with the weedy allele and have not been previously reported in the literature.

Each of the four putative resistance QTL contained at least one functionally characterized fungal resistance gene; however, all of them were annotated as blast resistance genes. Similarly, the major sheath blight resistance QTL *qShB9–2* in an indica rice variety Jasmine 85 was mapped at SSR marker RM245 (Liu et al. 2009). The blast resistance QTL *qBLAST9.3* in Jasmine 85 was mapped between SSR markers RM107 and RM245 (Jia and Liu 2011) suggesting that genomic region at or nearby RM245 may harbor genes important for both rice blast and sheath blight disease resistance. It is possible that some of these genes play a more general role in fungal resistance, but further studies are required to determine whether these genes or previously uncharacterized loci occurring nearby are responsible for the observed effects on ShB resistance.

Notably, none of these loci were identified with the data from the 2016 field season. This result suggests that there are strong environmental effects acting on ShB, as has been previously reported (Zou et al. 2000; Channamallikarjuna et al. 2010). Our inability to detect ShB resistance QTL in 2016 could partially be due to lower overall levels of phenotypic variation in ShB resistance compared with 2015. This is possibly due to fewer of the RILs showing highly infected scores (e.g., > 5) (see Fig. 1 b and d). The phenotypic distributions in 2016 were also bimodal, which was consistent with our observation that one QTL, controlled by *sd1*, explained the largest proportion of the variance. This phenotypic difference between years suggests that the environmental conditions leading to genotype-specific ShB susceptibility in 2015 were not present in 2016. The 2016 field season experienced extensive rainfall, which resulted in the lodging of some plants during the disease evaluation period. It is possible that this introduced additional phenotypic variance which impaired our ability to map traits from that season.

### Utilizing Phenotypic Covariates to Map ShB Resistance QTL

Previous studies of ShB resistance QTL have struggled to deal with the effects of PH and HD, and the common methods employed to deal this issue have had serious drawbacks (Zeng et al. 2015). By including these confounding traits as covariates in our mapping models we were able to overcome these issues. The primary method for dealing with PH and HD in the literature is to ignore any QTL that co-localizes with a PH or HD QTL (Zeng et al. 2015). This method is flawed because it requires that the correlated QTL have previously been identified and that a correlated QTL does not co-localize with a resistance QTL by chance. This is a serious problem considering that PH and HD QTL with large confidence intervals can be found across the whole rice genome. By measuring these traits alongside ShB resistance and including them as covariates, we can be more confident that the remaining QTL are actually involved in ShB resistance.

Another common method is to choose parents for the mapping population that are similar in PH and HD (Liu et al. 2013). This has two potential downsides. First, these traits could be polygenic and show transgressive segregation in the mapping population. This would result in a population that still shows strong correlations between ShB resistance and plant growth traits despite the parental similarity. The second downside is that it limits the potential for creating diverse mapping populations if only plants of similar growth characteristics can be chosen as parents. By including confounding plant growth traits as covariates in our mapping models, it is possible to map ShB resistance QTL using crosses between parents with drastically different morphological characteristics. Despite the morphological differences between the parents of our populations, we were still able to map multiple putative ShB resistance QTL. The fact that three of our four QTL increased resistance with the weedy allele indicates that these crop **×** weed crosses can be valuable tools for breeding purposes and are an underutilized source of novel genes.

One potential caveat to note in our analyses is that the PH and HD data were collected in the 2012 field season, while ShB resistance was measured in 2015 and 2016. Despite the fact that the same lines were used in all three growing seasons, it is possible PH and HD may vary substantially with year and confound mapping. This is unlikely, however, as both traits are highly heritable (Han et al. 2017). Additionally, the large percentage of the variance in ShB resistance that they can explain and the fact that their use as covariates masks known PH and HD QTL implies that their inclusion in our model is appropriate. It is also possible that *R. solani* infection has an impact on PH (i.e. that highly susceptible plants are shorter due to negative effects of infection). Because we wanted to test how PH impacts ShB resistance, not how ShB resistance impacts PH, it was important to collect PH data in the absence of fungal infection.

### Implications for Breeding and Variety Selection

Our observation that the difference in ShB resistance between cultivated and weedy rice is mostly due to PH and HD has some implications for the disease management in the region. It is possible that by choosing taller crop varieties with later heading dates, rice farmers may be able to increase their yield in fields chronically affected by sheath blight and weedy rice. Despite the large number of QTL identified in previous studies, few if any have been utilized in breeding programs because they tend to have small effects (Zeng et al. 2015). It has been suggested that breeding programs should stop waiting for large-effect loci to be discovered and instead begin breeding lines that pyramid multiple small-effect loci in a single genetic background. Utilizing an approach that emphasizes growing varieties with favorable plant architectures and multiple small to moderate-effect QTL, such as those identified in this study, is likely the best way to combat ShB. The blast resistance genes occurring within the ShB resistance QTL identified here and previously may be good candidates for future studies (Liu et al. 2009; Jia and Liu 2011). Next steps in this system include fine mapping with near isogenic lines and functional verification of candidate genes.

## Conclusions

We identified four putative ShB resistance QTL that were not associated with PH or HD, two of which have not been reported in the literature. These QTL can be used in combination with other small to moderate effect resistance QTL to breed for more disease resistant rice varieties. Additionally, our approach of using PH and HD as covariates in our mapping models can be a powerful tool for identifying ShB resistance QTLs in future studies.

## Supplementary information

**Additional file 1: Table S1.** Functionally characterized genes from the QTARO database. Candidate genes for each QTL are highlighted in yellow.

**Additional file 2: Table S2.** Phenotyping data for the B population including ShB susceptibility in 2015 and 2016, PH in 2012, and HD in 2012. (CSV 4 kb)

**Additional file 3: Table S3.** Phenotyping data for the S population including ShB susceptibility in 2015 and 2016, PH in 2012, and HD in 2012. (CSV 3 kb)

**Additional file 4: Table S4.** The genotypes of the top 10% most resistant RILs in the 2015 season at each of our putative QTL.

**Additional file 5: Table S5.** Genotyping data of RILs in the B population generated using genotyping-by-sequencing in the F_5_ generation. (CSV 5483 kb)

**Additional file 6: Table S6.** Genotyping data of RILs in the S population generated using genotyping-by-sequencing in the F_5_ generation. (CSV 1736 kb)

## Data Availability

All data generated or analyzed during this study are included in this published article and its supplementary information files.

## Abbreviations

BHA: black hull awned
DGWG: Dee-Geo-Woo-Gen
HD: heading date
LOD: logarithm of odds
PDA: potato dextrose agar
PH: plant height
QTL: quantitative trait locus
RIL: recombinant inbred line
SH: straw hull
ShB: Sheath blight
SNP: single nucleotide polymorphism
